# Cystinosis Presenting with Findings of Bartter Syndrome

**DOI:** 10.4274/jcrpe.v3i2.21

**Published:** 2011-06-08

**Authors:** Behzat Özkan, Atilla Çayır, Celalettin Koşan, Handan Alp

**Affiliations:** 1 Atatürk University, Department of Pediatric Endocrinology, Erzurum, Turkey; 2 Atatürk University, Department of Pediatric Nephrology, Erzurum, Turkey; 3 Atatürk University, Department of Pediatrics, Erzurum, Turkey

**Keywords:** Cystinosis, bartter syndrome, metabolic alkalosis

## Abstract

A five-year-old boy was referred to our pediatric clinic for evaluation of failure to thrive, headache, intermittent high fever, restlessness, polyuria, and polydipsia. His weight and height measurements were under the 3rd percentile. Clinical findings consisted of frontal bossing, carious teeth, O-bain deformity of the lower extremities, and moderate dehydration. The presence of metabolic alkalosis, hypokalemia, hypochloremia, and high renin and aldosterone levels were suggestive of Bartter syndrome and a treatment regimen for Bartter syndrome was started.  At follow-up, the polyuria and hyponatremia were found to persist. A reassessment of the patient revealed findings consistent with proximal renal tubular acidosis such as metabolic acidosis with a high urinary pH, proteinuria, aminoaciduria with phosphaturia and hypercalciuria. Based on the presence of parental consanguinity as well as polyuria, proteinuria, low tubular reabsorption of phosphorus, generalized aminoaciduria, light yellow skin and hair color, the probable diagnosis of cystinosis was established and was confirmed by slit-lamp examination of the cornea showing cystine crystal deposition. Our case is a good example demonstrating that development of metabolic alkalosis does not exclude cystinosis and that all findings of the patient should be thoroughly evaluated.

**Conflict of interest:**None declared.

## INTRODUCTION

Cystinosis is caused by a defect in transport of cystine across the lysosomal membrane, due to defective function of the lysosomal membrane protein cystinosin, resulting from mutations of the cystinosin gene (CTNS) (1).  This gene resides on chromosome 17p13 and has 12 exons, the last 10 of which code for cystinosin. There are three variants of clinically recognizable cystinosis, namely, the infantile (nephropathic) form, the juvenile (intermediate) form and the adult (benign) form (2,3). The infantile nephropathic cystinosis is a rare form occurring in 1:100 000-200 000 of live births (4). The defective transport of cystine across the lysosomal membrane causes an accumulation of this aminoacid in cells, affecting the functions of organs such as kidneys, eyes and the thyroid gland (3,4,5). Symptoms in nephropathic cystinosis appear within the first 3-12 months of life. These patients typically present with symptoms of severe fluid and electrolyte disturbance, renal Fanconi syndrome, vitamin D-resistant rickets, metabolic acidosis, growth failure, and photophobia. The clinical state progresses to end-stage renal failure (ESRD) by the end of the first decade of life if left untreated (6,7). This report describes a child who presented with growth retardation and was initially diagnosed as Bartter syndrome, but subsequently as cystinosis. 

## CASE REPORTS

A five-year-old boy was referred to our department for evaluation of failure to thrive, intermittent high fever, restlessness, polyuria, and polydipsia. The patient was born at term with a birth weight of 3500 g. Pregnancy and delivery were reported as uncomplicated. There was no history of persistent diarrhea and vomiting. The parents were first-degree relatives.   The patient was admitted for further evaluation and treatment of his metabolic alkalosis, polyuria and hypokalemia. On physical examination, his weight was 11 kg (<3rd  percentile) and his height was 83 cm (<3rd percentile). Blood pressure was 90/60 mmHg (normal for age), pulse rate 104 beats/min, and temperature 36.6°C. He had blond hair and fair skin and a sluggish appearance. Frontal bossing, carious teeth, and O-shaped deformity of the lower extremities were noted. Signs of moderate dehydration such as dry oral mucosa, sunken eyes, and decreased skin turgor were present. Neurological and mental status evaluations were reported as normal.

Whole blood count was normal (Hb 12 g/dL, Htc 37%, WBC 9,600/mm3, platelets 185 000/mm3). Serum sodium level was 118 mEq/L, potassium 2.0 mEq/L, chloride 78 mEq/L.  Blood urea nitrogen was 32 mg/dL, creatinine 1.4 mg/dL, calcium 9.3 mg/dL, phosphorus 2.7 mg/dL, alkaline phosphatase 400 U/L, ALT 32 U/L, AST 28 U/L and glucose 93 mg/dL. Urinalysis showed a specific gravity of 1002 and pH level of 7.0. Glucosuria (+)  and proteinuria (++) were present. Microscopic examination of the urine sediment was normal. Venous blood gas analysis revealed values consistent with metabolic alkalosis (pH 7.51, pCO2 33 mm Hg, bicarbonate 28.8 mmol/L). Twenty-four-hour urine volume was 4200 mL/m2 /day (7.5 mL/kg/day), indicating polyuria. Tubular reabsorption of phosphorus was 78% (90-110%).  Twenty-four-hour urinary calcium excretion was 12.5 mg/kg (normal rate: <4 mg/kg/day). Blood renin and aldosterone levels were increased (66 mg/mL and 1020 ng/dL, respectively). Generalized aminoaciduria was detected. 

**Figure 1:**
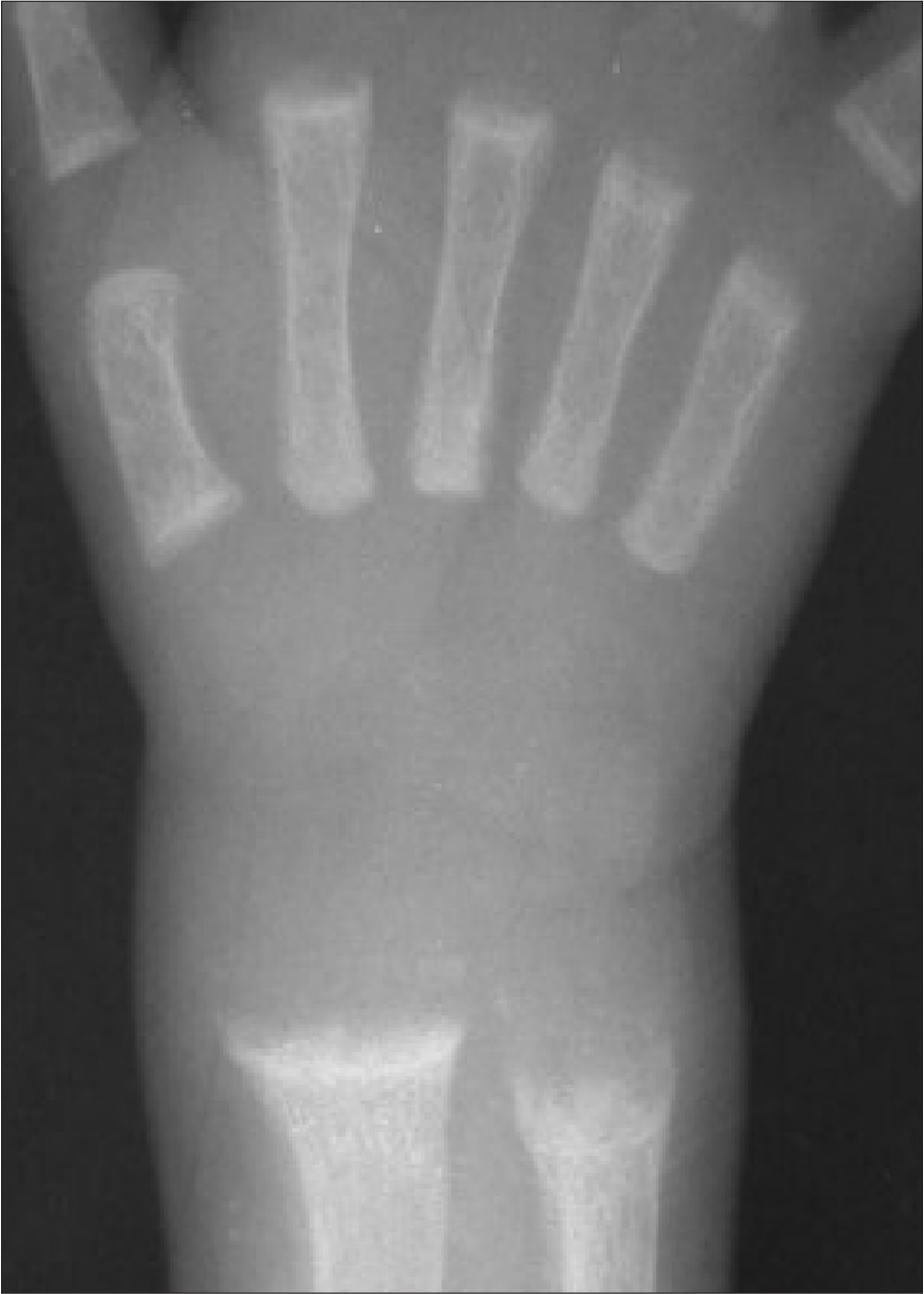
The metaphyses are frayed, cupped and splayed in the distal radius and ulna in rickets

Based on the presence of metabolic alkalosis, hypokalemia, hyponatremia, hypochloremia and high renin and aldosterone levels, a diagnosis of Bartter syndrome was made and the patient was started on a treatment regimen consisting of sodium chloride, potassium citrate, indomethacin, hydrochlorothiazide and amiloride. At this time, the sweat chloride level was normal (34 mEq/L). Radiologic examination of the left wrist showed active rachitic changes. The serum level of 25(OH)D was 9.3 ng/mL (<11 ng/mL) and an anterio-posterior X-ray of the left wrist was consistent with rickets (Figure 1). Renal ultrasonography findings were normal. Calcitriol treament in a dose of 25 μ/day was started. 

**Figure 2:**
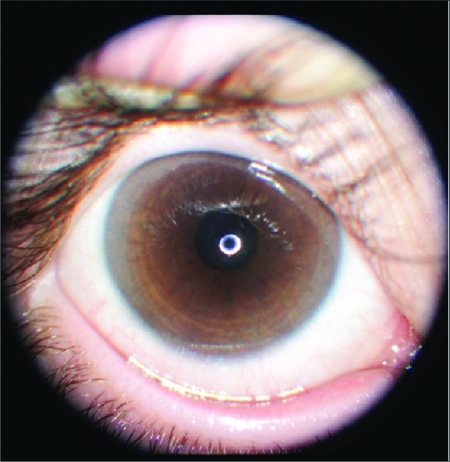
Slit-lamp examination of the cornea showing cystine crystal deposits

One month later, the patient developed metabolic acidosis with a high urinary pH (pH 6). Presence of proteinuria, aminoaciduria, glucosuria, phosphaturia and hypercalciuria was suggestive of proximal renal tubular acidosis. Blood renin and aldosterone levels were retested and found to be normal. The treatment schedule was changed to a combination of sodium bicarbonate, hydrochlorothiazide-amiloride, potassium citrate, indomethacin, Joule’s solution and Shohl’s solution. An overall evaluation of the clinical and laboratory findings of the patient led to a probable diagnosis of cystinosis. The diagnosis was confirmed by slit-lamp examination of the cornea showing cystine crystal deposits (Figure 2). 

Thyroid function tests were within normal range. Renal ultrasonography showed both kidneys to be normal. The  patient is at present being treated with 1,25-dihydroxycholecalciferol, sodium bicarbonate, potassium, Shohl’s solution, Joule’s solution, cysteamine (Cystagon  50 mg/kg per day in four doses) and carnitine. The patient gained 1200 g in weight within one month of the initiation of this regimen. The polyuria has improved and serum electrolyte levels have returned to normal.

## DISCUSSION

Cystinosis is a genetic disorder of autosomal recessive inheritance due to defective lysosomal cystine transport leading to excessive intracellular cystine accumulation. The proximal tubule is very sensitive to cystine storage and these patients manifest the features of the Fanconi syndrome. The typical clinical picture is renal Fanconi syndrome in most infants with cystinosis (6,7). In this form of the classic childhood nephropathy, patients appear clinically normal at birth. However, depending on the degree of the disease, tubulopathy characterized by metabolic acidosis, glucosuria, aminoaciduria, bicarbonaturia, phosphaturia, hypokalemia develops in the first decade of life and these signs are mostly consistent with renal Fanconi syndrome. The typical presentation of these patients is proximal renal tubular acidosis (7,8). However, some patients diagnosed as cystinosis may present initially with metabolic alkalosis (8,9,10,11,12,13). In our patient, although the initial laboratory findings were suggestive of renal tubular Fanconi syndrome, the levels of blood gases were compatible with metabolic alkalosis. The patient’s serum renin and aldosterone levels were above normal, serum sodium and chloride levels were low, metabolic alkalosis was present. Excretion of urinary sodium and chloride was increased. Based on these findings, Bartter syndrome was the initially considered diagnosis. During follow-up, in a polarized eye examination made for the evaluation of the patient's photophobia, cystine crystals were detected. Approximately one month later, the nature of the levels of the blood gas composition changed and became compatible with acidosis (pH: 7.2; HCO3: 10 mEq/L; pCO2: 25 mEq/L) Urine pH was high. The reason why cystinosis presents with metabolic alkalosis, simulating the findings of Bartter syndrome, is still unknown. Tubular dysfunction resulting from cystine accumulation may cause these findings, but the pathogenesis of intralysosomal cystine accumulation leading to the dysfunction of the proximal tubule has not yet been resolved. Yildiz et al (13) reported a 16-month-old male patient presenting with signs of Bartter syndrome. This patient also, later on, was diagnosed as cystinosis.  To explain these findings, the authors speculated that a sodium-dependent transtubular transport defect causes increased distal tubular delivery of sodium, which in turn leads to an enhanced exchange of sodium for potassium and hydrogen ion, perpetuating a contraction alkalosis. In some studies, hyperplasia of the juxtaglomerular apparatus with elevated levels of renin and aldosterone due to hyponatremia was reported in patients with cystinosis and these findings were attributed to an increased exchange of sodium for hydrogen and potassium ion caused by the hyperreninemia and hyperaldosteronism (8,13). In our patient, serum sodium level was low. Renin and aldosterone levels were above normal initially and decreased to normal on follow-up.  

A definite diagnosis of cystinosis can be made by showing increased levels of cystine in leukocytes or fibroblasts (14). The diagnosis can also be reached by demonstration of cystine crystals in tissues such as bone marrow, lymph nodes, or conjunctiva (14,15). In our patient also, the final diagnosis was made by showing cystine crystals in the ocular tissue. Cysteamine therapy constitutes the mainstay of treatment of cystinosis, supported by measures to improve water-electrolyte and acid-base disturbances. In some cases, indomethacin is used to reduce the loss of water, sodium and potassium by the renal tubules. Vitamin D is used to correct the hypocalcemia and carnitine therapy is instituted to keep the reduced levels of carnitine within the normal range. 

In conclusion, as also demonstrated in our patient, cystinosis can initially present with the laboratory findings of Bartter syndrome. New studies are needed to clarify the etiopathogenesis of this association. 
